# Global perspectives of determinants influencing HPV vaccine introduction and scale-up in low- and middle-income countries

**DOI:** 10.1371/journal.pone.0291990

**Published:** 2024-01-16

**Authors:** Dominique Guillaume, Dur-e-Nayab Waheed, Meike Schleiff, Kirthini Kasi Muralidharan, Alex Vorsters, Rupali J. Limaye

**Affiliations:** 1 International Vaccine Access Center, Johns Hopkins Bloomberg School of Public Health, Baltimore, Maryland, United States of America; 2 Jhpiego, A Johns Hopkins University Affiliate, Baltimore, Maryland, United States of America; 3 Center for Infectious Disease and Nursing Innovation, Johns Hopkins University, Baltimore, Maryland, United States of America; 4 Center for the Evaluation of Vaccination, University of Antwerp, Antwerp, Belgium; 5 HPV Prevention and Control Board, University of Antwerp, Antwerp, Belgium; 6 Department of International Health, Johns Hopkins Bloomberg School of Public Health, Baltimore, Maryland, United States of America; 7 Department of Health, Behavior & Society, Johns Hopkins Bloomberg School of Public Health, Baltimore, Maryland, United States of America; 8 Department of Epidemiology, Johns Hopkins Bloomberg School of Public Health, Baltimore, Maryland, United States of America; University of Liverpool & International Livestock Research Institute (ILRI), UNITED KINGDOM

## Abstract

Achieving WHO cervical cancer elimination goals will necessitate efforts to increase HPV vaccine access and coverage in low-and-middle-income countries (LMICs). Although LMICs account for the majority of cervical cancer cases globally, scale-up of HPV vaccine programs and progress toward coverage targets in LMICs has been largely insufficient. Understanding the barriers and facilitators that stakeholders face in the introduction and scale-up of HPV vaccination programs will be pivotal in ensuring that LMICs are equipped to optimize the implementation of HPV vaccination programs. This qualitative study interviewed 13 global stakeholders categorized as either academic partners or global immunization partners to ascertain perspectives regarding factors affecting the introduction and scale-up of HPV vaccination programs in LMICs. Global stakeholders were selected as their perspectives have not been as readily highlighted within the literature despite their key role in HPV vaccination programming. The results of this investigation identified upstream (e.g., financial considerations, vaccine prioritization, global supply, capacity and delivery, and vaccine accessibility, equity, and ethics) and downstream (e.g., vaccine acceptability and hesitancy, communications, advocacy, and social mobilization) determinants that impact program introduction and scale-up and confirmed that strong political commitment and governance are significant in garnering support for HPV vaccines. As LMICs introduce HPV vaccines into their national immunization programs and develop plans for scaling up vaccination efforts, strategic approaches to communications and advocacy will also be needed to successfully meet coverage targets.

## Introduction

Cervical cancer is the fourth most frequently diagnosed cancer among women globally and is the leading cause of cancer-related deaths among women living in low-and-middle-income countries (LMICs). LMICs experience the largest global burden of cervical cancer, with 84% of new cases and 90% of cervical cancer-related deaths occurring in LMICs [1–3]. The primary etiology of cervical cancer is infection with the human papillomavirus (HPV). Biomedical advances such as the development of the HPV vaccine have made cervical cancer largely preventable. The HPV vaccine has been commercially available for more than a decade and protects against high-risk strains of HPV that cause approximately 91% of all cervical cancer cases [4, 5]. Although LMICs experience the highest burden of cervical cancer, access to HPV vaccines in these countries remains inadequate. As of 2021, while more than 85% of high-income countries (HICs) had introduced HPV in their national immunization schedules, less than 25% of low-income, less than 30% of lower-middle-income, and less than 60% of upper- middle-income countries had done so [6].

The WHO global strategy for cervical cancer elimination has established 90-70-90 targets, which aim to fully vaccinate 90% of girls against HPV by 15 years of age, screen 70% of women for cervical cancer, and ensure that 90% of women who are diagnosed with pre-cancerous lesions receive treatment and care [7, 8]. Vaccination is the cornerstone of this elimination strategy, as recent landmark studies have reported that HPV vaccines reduced cervical cancer incidence by nearly 90% over a 10-year period [9–13]. LMICs experience significant challenges in the introduction and scale-up of HPV vaccines, which contributes to suboptimal coverage rates [14, 15].

As of 2022, 27.6% of low-income countries, 43.1% of low-and-middle-income, and 63.9% of upper-middle-income countries have introduced HPV vaccines at either a sub-national or national level [16]. These numbers are in stark contrast to high-income countries, where 90% of high-income countries have introduced HPV vaccines [16]. According to the WHO, current estimates for global coverage with the first dose of HPV among adolescent girls is estimated at 15% which is a proportionally significant reduction from 20% in 2019 [17]. HPV vaccine introduction, coverage, and scale-up in LMICs is impacted by a range of factors including health system constraints, patient barriers, policy barriers, and challenges related to the COVID-19 pandemic and its impacts on health priorities within countries [12, 13, 18, 19]. Global stakeholders such as Gavi, WHO, and UNICEF, among others, have been instrumental in increasing access to HPV vaccines within LMICs through providing monetary and technical assistance support [20, 21]. Additionally, academic institutions have been at the forefront in leading research and generating essential data needed by stakeholders and policy-makers to inform the introduction of HPV vaccines [22, 23]. While research has been conducted evaluating factors influencing HPV vaccine introduction in LMICs, these studies have focused primarily on obtaining national stakeholder and community perspectives, with a lesser focus on obtaining global stakeholder perspectives, namely perspectives from academic institutions and international stakeholders and organizations [24–26]. This study aimed to address this gap by exploring global stakeholder perspectives regarding determinants affecting HPV vaccine introduction and scale-up in LMICs through key informant interviews.

## Methods

### Stakeholder identification

Our manuscript focused on obtaining data from global stakeholders as their perspectives are not as readily highlighted in the literature. The majority of studies that explore challenges in the HPV vaccination landscape in LMICs largely focus on obtaining data from national stakeholders (e.g. Ministries of Health), local stakeholders (e.g. health care providers, program implementers, teachers) and beneficiaries (e.g. adolescents, parents) [24, 27, 28]. The perspectives of global stakeholders are crucial to highlight as the financial and technical support from global partners has been influential in facilitating country decisions to introduce HPV vaccines, and the implementation of vaccination programs [22].

Global stakeholders were categorized into two groups: academic partners and global immunization partners. Academic partners were defined as individuals working at post-secondary institutions with research expertise in HPV vaccine program implementation in LMICs. Academic partners were identified through a targeted search of scientific databases containing peer-reviewed publications related to HPV vaccine programming in LMICs. In addition, a grey literature search was conducted to identify stakeholders who had published relevant non-peer-reviewed literature (e.g., programmatic reports, abstracts, dissertations). Academic partners were also identified through membership databases of pertinent professional organizations (e.g., International Papillomavirus Society). While we prioritized academic partners based in LMICs, we also included academics based in HICs who had research experience relevant to HPV vaccine program introduction, implementation, evaluation, and/or scale-up in LMICs.

Purposive sampling was used to select participants. We sought to include participants with relevant knowledge and experience in HPV vaccine programming in LMICs [29]. Snowball sampling was also employed, in which selected participants within each stakeholder group referred other potential participants. The research team sent potential participants emails explaining the study aims and procedures.

Global immunization partners were defined as individuals working in multilateral or bilateral organizations (e.g., WHO, Gavi, International Agency for Research on Cancer, United Nations International Children’s Emergency Fund [UNICEF], Bill and Melinda Gates Foundation [BMGF]) with expertise in HPV vaccine program implementation in LMICs. To identify global immunization partners, we generated a list of multilateral and bilateral global health organizations, and selected organizations with active programs focused on HPV vaccination in LMICs. Stakeholders affiliated with these organizations were eligible for inclusion as global immunization partners if they had an active role or technical expertise in HPV vaccination programs within LMICs.

### Data collection

To create semi-structured interview guides, we reviewed the literature on challenges and facilitators to vaccine introduction and scale-up in LMICs, as well as general information related to HPV vaccination programs in LMICs. Interview guides included questions related to 1) challenges to HPV vaccine introduction and scale-up, 2) vaccine prioritization, 3) vaccine delivery, 4) vaccine hesitancy, and 5) scale-up of HPV vaccines during the COVID-19 pandemic. Questions were open-ended with probes to explore participant perspectives [30]. Questions were pre-tested with individuals with experience in HPV vaccine introduction and scale-up in LMICs. Interviews were conducted between January 2022 –June 2022. Interviews were conducted in English over Zoom video conferencing software. All interviews were audio recorded. Transcripts were reviewed and edited for clarity and accuracy.

### Data analysis

Data analysis was performed using qualitative content analysis. Two transcripts were randomly selected for open coding by two authors, and a code list was subsequently developed. The two authors then coded two additional transcripts to refine the code list. Additional rounds of open coding were conducted until no new codes emerged, and the two authors agreed on the code list. Codes were generated inductively, and authors discussed the codes and agreed on the codebook. Patterns among the codes were identified and the codes were then categorized into broader themes. The codebook structure consisted of codes, their corresponding themes, a brief definition of each code, guidelines for when to use each code, and corresponding example passages. The coding framework was subsequently applied to all remaining transcripts [31, 32]. The two authors met regularly throughout the coding process. After coding was complete, the broader research team met to discuss and agree upon emerging themes.

### Ethics

This study was reviewed and given an exemption by the Johns Hopkins Bloomberg School of Public Health Institutional Review Board (IRB00019084). While this study was characterized as exempt, we explained the study aims and procedures with each participant and allowed the participant to ask any clarifying questions prior to conducting interviews.

## Results

**Fig 1:**
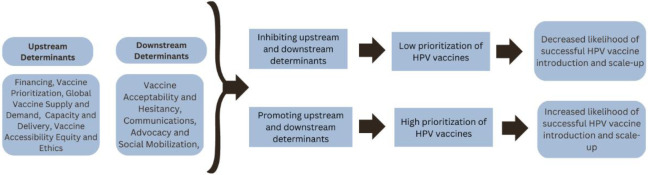
Determinants influencing HPV vaccine introduction and scale-up.

We contacted 61 stakeholders (45 academic partners and 16 global immunization partners). Global immunization partners were based in the following WHO regions: Africa (WHO AFR), South-East Asia Region (SEAR), Americas (AMR), and European Region (WHO EUR). Academic partners were based in 17 countries spanning sub-Saharan Africa (Kenya, Zambia, Rwanda, South Africa, Ethiopia, Mozambique, Botswana, Cote d’Ivoire, Nigeria, Malawi), Southeast Asia (Cambodia, Lao PDR, Indonesia, India), Australia, the US, and the UK. Of the 61 stakeholders contacted, 20 were interested in participating in an interview, and a total of 13 interviews were completed. Participating academic partners represented five countries and participating global immunization partners represented five WHO regions (Table 1). The themes that emerged during interviews were categorized into upstream and downstream determinants of HPV vaccine introduction and scale-up (Fig 1).

**Table 1:** Stakeholder characteristics.

**Academic Partners** (n = 7)	**Country**	**Expertise**
	South Africa	Vaccinology
	South Africa	Vaccinology
	Kenya	Obstetrics and Gynecology
	UK*	Vaccine epidemiology
	UK*	Economics and health systems
	Zambia	Health promotion and public policy
	Nigeria	Health promotion
**Global Immunization Partners** (n = 6)	**Region**	**Expertise**
WHO	AFR, EUR	Vaccine preventable diseases and immunization
UNICEF	AFR	Health promotion
GAVI	AFR	Global health policy
BMGF	AFR	Health policy and finance, public health economics
IARC	AFR, EUR, AMR	Obstetrics and Gynecology, cancer prevention
PATH	AFR, SEAR, WPR	Immunization programs

*Participants based in the UK had sustained programs of research in LMICs

### Upstream determinants to introduction and scale-up

Upstream determinants were defined as macro-level factors that impacted program introduction and scale-up and included financial considerations, vaccine prioritization, global supply, capacity and delivery, and vaccine accessibility, equity, and ethics (Table 2).

**Table 2:** Characterization of upstream and downstream determinants.

Determinant	Definition	Promotion of Determinants	Inhibition of Determinants	Illustrative Quotes Quotes
**Upstream Determinants**				
**Financing**	Financial considerations (e.g. vaccine pricing, cost of delivery, economic burden, human resources, vaccine cost compared to screening, etc.) that can serve as facilitators or detractors to introduction or scale-up.	Availability of financing through global partners such as Gavi facilitates introduction.	Limited budgets decrease the prioritization of HPV vaccines and can influence delivery selection	“One of the greatest challenges is really in [financing]. You can look at this either on the supply side on the demand side. And on the supply side, one of the greatest issues has really been with having the financial capacity to be able to introduce the vaccine.” Academic Researcher
**Vaccine Prioritization**	Factors influencing stakeholder prioritization of HPV vaccines, including political commitment and buy-in, relevant data points, and vaccine impact.	The availability of strong data can endorse strong political commitment. This in turn accelerates vaccine prioritization and introduction.	The lack of political will and data serves as a deterrent for vaccine introduction and hinders the creation of immunization policy.	“If you just look at what other certain things in health that were deprioritized the past few years with COVID…the HPV program was already deprioritized…women and girls health has been deprioritize in the past two years. If you’ve got only one team trying to deliver multiple agendas, it makes sense that something’s got to drop.” Global Immunization Partner
**Vaccine Supply and Demand**	Availability of vaccines globally and how vaccine supply can influence subsequent vaccine introduction and scale-up in LMICs.	Countries are more likely to introduce and meet coverage rates with adequate supply.	Supply deficits cause significant delays to country introduction. For countries that have already introduced, limited supply can impact delivery strategies (i.e. single age cohort v.multi age cohort vaccination)	“… because of the global supply crisis, some of the LMICs withheld introduction of the vaccine for one year, I know some of the countries are waiting for more than two years. So that is another challenge…” Global Immunization Partner
**Capacity and Delivery**	The state of health systems, human resources, immunization programming and delivery, and monitoring and evaluation of program impact which can influence vaccine introduction and scale-up of HPV vaccines.	Sufficient capacity and effective delivery strategies to reach in-school and out-of school adolescent girls underpins reaching coverage targets.	Inefficient capacity to deliver vaccines (e.g. health system, human resources, cold chain) leads to demotivation for vaccine introduction and suboptimal coverage.	“We’re trying to ask countries to routinize, the HPV vaccine…that it becomes part of the routine program and is readily available. Due to the infrastructure and characteristics of the health system, it may be more optimal for them to deliver the HPV vaccine, like a campaign every few years, and just get all the girls at once.” Global Immunization Partner
**Vaccine Accessibility, Equity, and Ethics**	Access to HPV vaccines for communities at-large including vulnerable populations, and the right for individuals to be protected and equipped with information pertaining to vaccination including risks and benefits.	Enhancing accessibility, equity, and ethics contributes to promoting vaccine awareness and acceptability and reducing hesitancy.	Introducing vaccines without developing strategies to promote accessibility and equity contributes to low community buy-in resulting in low uptake.	“A lot of studies have been done within the African context, to understand the level of hesitancy when it comes to the HPV vaccination program. It’s difficult to actually measure to quantify…the challenge comes in can you really say that a population is hesitant to a vaccine that actually is not available in their context, when they don’t know about it, when there’s a lack of awareness?” Academic Researcher
**Downstream Determinants**				
**Vaccine Acceptability and Hesitancy**	Delays in the acceptance or refusal of vaccines despite the potential availability of vaccine services among communities.	Increasing acceptability through vaccine advocacy, dispelling misinformation, and providing platforms for knowledge dissemination reduces hesitancy.	The lack of strategies to mitigate hesitancy in vaccine introduction results in low uptake.	“I think the bigger problem we’ve been facing now is how social media can really push in a whole sorts of information that are not factual about vaccines. For some reason, I think people tend to be taken with more negative sentiments than positive sentiment…the issues still remain much bigger than maybe it’s being addressed.” Academic Researcher
**Communications, Advocacy, and Social Mobilization**	Efforts to disseminate information pertaining to HPV vaccines, and involve communities in developmental activities that support HPV vaccine introduction	Engagement with trusted leaders to promote advocacy and funding for communications efforts is instrumental to generating community acceptance.	Diminished funding for communications efforts can result in an increase of misinformation once vaccines are introduced.	“We are finding that over time as it seems that the social mobilization, this campaigning, and advocacy starts to dwindle, and that creates challenges. Because it encourages misinformation if the right information is not easily accessible to those who need to find them.” Academic Researcher

#### Financial considerations

All stakeholders identified funding and pricing as substantial challenges in HPV vaccine introduction and scale-up. Since 2012, Gavi has subsidized the introduction of HPV vaccines for girls ages 9–14 in LMICs. Gavi negotiated a vaccine price of approximately US $4.50 per dose for LMICs, which is significantly less than the market price [20, 33]. In Gavi’s co-financing model, eligible countries pay for a portion of the cost of Gavi-supported vaccines; as a country’s Gross National Product increases, it is responsible for an increasing share of the cost [34]. Although Gavi support is available for many countries, the HPV vaccine is relatively expensive: *“[Financial challenges have] changed over time*, *originally [it] was the cost*. *That’s why all the initial introductions were in high-income countries… those barriers [have] mostly been removed for low-income countries because Gavi now funds it*, *and the price that Gavi gets…I really wouldn’t say it’s low*, *but it’s a lot lower than it was before*.”- Academic Researcher

Stakeholders emphasized that HPV vaccine programs have additional costs that are substantial compared to traditional childhood vaccines, as these programs require extensive resources and capacity to reach the target population. These technical and financial requirements often result in suboptimal vaccine coverage as well as delays in scale-up: *“They were doing school programs*, *but that was expensive*, *because of [the number of] health care workers*. *In most areas*, *you have one healthcare worker manning a health center*. *That means the health center gets closed and other services are shut down*. *Number two*, *then you need to give the health care worker money and transport to go and vaccinate in the schools*. *So that’s a limitation because it’s clear across data school programs work better…”*-Academic Researcher

Limited budgets force countries to make difficult decisions and can lead to countries choosing other health programs that are less expensive and less resource-intensive than the introduction and scale-up of HPV vaccines: “*All these competing [health] issues also competing for a limited national health budget*. *And I think that’s where the greatest issue is*, *national governments are having to decide between malaria programs and HIV programs and TB programs*, *which are long-standing existing issues*, *and whether they are at a position to introduce the HPV vaccine*, *which sometimes falls a little lower in terms of the public health agenda*.”–Academic Researcher

Global immunization partners indicated that some countries that had introduced HPV vaccines with Gavi support found it extremely difficult to keep up with their co-financing. Gavi support helped with the financing and procurement of vaccines, but stakeholders described how countries struggled to allocate a portion of their national health budget for HPV vaccination programs: *“In terms of introduction*, *I think the biggest obstacle is the cost of the vaccine*, *even for Gavi countries*, *because it’s the most expensive vaccine in the EPI portfolio… So*, *especially low-income countries find it difficult to fulfill the Gavi copayment*.*”*–Global Immunization Partner

For countries transitioning out of Gavi support, cost-related challenges are a huge barrier for scale-up, as governments become responsible for the entire vaccine cost. Stakeholders voiced concerns regarding the sustainability of these vaccination programs, with more attention needed in Gavi-transitioning countries. Similar sentiments were expressed for middle-income countries that do not qualify for Gavi support and have to self-finance for the vaccine: *“I think the group that have struggled have been the middle-income countries like South Africa*, *like Mexico… countries that are not going to be eligible but have big populations who would benefit and where cervical cancer is a problem but [the countries] have to fund it themselves*.*”*–Academic Researcher

#### Vaccine prioritization

Political commitment and buy-in are critical in prioritizing the introduction and scale-up of HPV vaccines. Several stakeholders mentioned Rwanda and Zambia as examples of how strong political will can bolster the successful introduction and scale-up of HPV vaccines. In Rwanda, the government prioritized women’s health issues. Similarly, in Zambia, increased political commitment from the First Lady helped to prioritize women’s health concerns like cervical cancer prevention within the Ministry of Health. One stakeholder described political will in Rwanda: *“I think there was an awareness of it [cervical cancer] as a sort of global problem*. *Rwanda is one of the countries of Africa where there’s a parity [seen] between men and women*, *you’ll hear about how actually*, *it’s a country with the highest percentage of women in Parliament at 60-something percent*. *So*, *I’m not super surprised that women’s health issues are prioritized*.*”* Academic Researcher. Commenting on antigen prioritization, one interviewee described how the lower status of women in society resulted in a lack of prioritization towards cervical cancer in certain instances: *“Why is this [cervical cancer] neglected*? *Why isn’t it getting the traction that it needs*? *You’re talking about women*, *we’re talking about that woman in the village*, *we’re talking that woman in the city*. *How do we put the value of women’s lives back on the table*? *So that resources are put in place to protect them when scientific knowledge is available*?*”*-Academic Researcher

Another stakeholder explained how certain political leaders in Zambia were instrumental to HPV vaccine introduction: *“The Ministry of Health really puts a lot of priority on the HPV vaccine which actually helped politically… it was a former president… the wife actually was an OBGYN*, *medical doctor… she was a specialist in obstetrics and gynecology*. *So*, *as the First Lady advocated for interventions that actually really try to elevate cervical cancer and HPV vaccine… I think the political will that came with that was the first lady was able to talk like that*, *really help the government to push a certain dose*, *at least in political corridors*.”—Academic Researcher

To enhance political buy-in, stakeholders need access to relevant data points (e.g., local disease burden, cost data, vaccine efficacy and safety), particularly in the context of competing health priorities. Several examples were given of various Ministers championing HPV vaccines due to their gender or professional background. In countries without strong support or political commitment for cervical cancer or general women’s health issues, HPV vaccine programs were not prioritized. One participant discussed political will in Kenya: *“We’d say [political commitment is] amazingly important because your politicians can also undermine your program*. *So*, *when they promote it [HPV vaccines] in the community*, *it makes a difference*. *… in Kenya right now… the only thing in politicians’ mind is the politics of our upcoming election*. *I don’t know if you can get their attention on other issues*, *or health matters*.*”—Academic Researcher*

Many LMICs lack data demonstrating vaccine impact. This poses a barrier to the introduction of HPV vaccines, as stakeholders were interested in seeing data on the vaccine’s long-term impact on disease burden and cost savings before advocating for introduction: *“What is the expected impact and how long it will take to see this impact*? *Because cervical cancer takes decades to develop*. *Unlike the other infant vaccines*, *it’s impossible to see an impact in a very short period of time*, *so there are many issues to overcome*.”–Global Immunization Partner

Another participant also mentioned the importance of being able to show vaccine impact: *“[What’s] been missing from LMICs is demonstration of impact*. *You know*, *there are very few… Last year*, *there was that publication that showed that after 10 years of HPV vaccination*, *they’re seeing declines in cervical cancer*. *It generated a lot of public interest*.”—Academic Researcher

Several participants voiced that visualizing the long-term impact of HPV vaccination programs is difficult, as cervical cancer is a slow-progressing disease. This is an additional barrier to generating buy-in and political will among key stakeholders: *“I think initially*, *HPV was a harder vaccine to make the case for simply because*, *with the other new vaccines that became available roughly the same time as other vectors like rotavirus*, *or even pneumo (PCV) vaccines*. *And with those vaccines*, *you introduced the vaccine this year*, *and within two or three years*, *you see a reduction in kids dying of diarrhea or pneumonia and so forth*. *Whereas with HPV*, *you’re telling people we’ll give it now and wait 20 years’ time… So it’s a big sell for governments who might not even be the same party in power in 20 years’ time… it’s a harder sell*…”–Academic Researcher

#### Global vaccine supply and demand

Given the global demand for HPV vaccines, supply challenges significantly hinder introduction and scale-up. Both academic stakeholders and global immunization partners noted that the supply of HPV vaccines has been inadequate as demand has increased globally. In certain instances, supply-related challenges were more significant than funding challenges, with stakeholders expressing that supply issues had substantially increased throughout the COVID-19 pandemic: *“I think that other challenges can be related to the availability of the vaccine in terms of the recent stock-outs that were experienced… because Nigeria was ready to introduce the vaccine*. *But unfortunately*, *there was a stock-out*, *and so they had to postpone*.*”*–Academic Researcher

Due to these supply challenges, some participants emphasized the feasibility of local vaccine production. Stakeholders noted the importance of new manufacturers entering the market and expanding local manufacturing capacity: *“I think that there’s also room for LMICs*, *and sub-Saharan African countries more specifically*, *to start to consider increasing their local manufacturing and production capacity to include generic HPV vaccination programs*. *The high levels of vaccine inequity that we’ve experienced with the COVID-19 vaccine has encouraged more of our countries to consider scaling up their vaccine manufacturing capacities*, *and hopefully*, *the HPV vaccine is within that agenda*. *And this will probably meet some of the needs that we have in our setting in terms of access to the vaccine at an affordable cost*.*”*–Academic Researcher

#### Capacity and delivery

Stakeholders mentioned several capacity considerations, including the strength of the health system, human resources, immunization programming and delivery, and monitoring and evaluation of program impact.

#### Health systems

The need for stronger health systems was an important factor influencing scale-up of HPV vaccines, including the capacity of health systems capacity to support introduction. Stakeholders described how health systems must have the necessary resources in place to not only adopt HPV immunization programs, but also sustain them over time. Governments rely heavily on external donor funding, but this funding often covers only the procurement of vaccines and not technical support, which is the most expensive aspect of these programs. An academic researcher referred to limited health systems capacity as a barrier: *“The HPV vaccination program is unlike routine infant immunization programs that the subcontinent has had long-standing experience with*. *It requires additional resources*, *additional capacity… It [the health system] needs to be ready*, *it needs to have the necessary resources to be able to not only adopt the HPV immunization program but also sustain it over time*. *And in some cases*, *unfortunately*, *in many of our sub-Saharan African countries*, *the status of our health systems is quite unstable*, *sometimes unpredictable*.*”* Stakeholders working in countries that had not introduced HPV vaccines into their national immunization schedules reported a need to strengthen national health insurance systems so that communities at-large could benefit and access HPV vaccines. This participant stressed the importance of a strong health insurance scheme: *“The health care system*, *and the kind of health care financing that we have*, *we don’t really have a robust health insurance scheme…yes*, *we have a national health insurance scheme*, *but still*, *it’s just an insignificant percentage of the entire population that benefits from it and even that is still not as optimal as you may wish*. *So*, *this system is a factor [in low HPV vaccine coverage] … there is no specific pathway*, *a single path we will say okay*, *this is how we assess*, *analyze and introduce*, *and that is strictly been adhered to*. *So that is one of the factors that determines whether or not it will be introduced*.”–Academic Researcher

Health systems in many LMICs are ill-equipped in successfully addressing adolescent health issues. This is partly attributable to health care providers not receiving sufficient training in providing care to adolescents, limited experience discussing sensitive issues regarding sexual and reproductive health, and provider stigma. One participant described the health system’s limited capacity to serve adolescents: *“Health systems are not responsive to the needs of adolescents*. *The HPV vaccine is often the first time a young person will be interacting with the health system again since infancy*. *How are adolescents involved in potentially co-designing programs and monitoring and evaluation of programs*? *What does that practically look like*? *And what impact does that have on a more responsive program*, *and consequently*, *coverage and finding impact because that’s what we’re always trying to aim for as high coverage…some of our contexts where we’ve introduced this vaccine*, *young people aren’t really empowered to speak up or out*…”—Global Immunization Partner

Stakeholders recommended integrating HPV vaccines with additional adolescent health services, particularly within schools: *“We can improve HPV vaccination programs and overall adolescent health if we deliver these services together*. *In most countries… here is some understanding that adolescents don’t just need the HPV vaccine*, *that they have other health needs*, *whether it’s sexual reproductive education*, *whether it’s contraception*, *whether it’s other adolescent vaccines*, *whatever the case may be*, *but there seems to be discord in terms of delivering these vaccines as a comprehensive adolescent health package*.”–Academic Researcher. One participant spoke about vaccine delivery experience in Rwanda in which adolescent health services were integrated: *“They tried to use that opportunity [HPV vaccines] as a way to deliver [additional] services…before [HPV vaccines] I don’t think the health workers were going into schools to talk about menstrual hygiene*, *or to talk about other things*. *Before they just didn’t really deal with that…so I think they [the Ministry of Health] saw this as an opportunity to start delivering those services*.*”* -Academic Researcher

Global stakeholders emphasized that human resources in many LMIC settings are insufficient, particularly in rural areas. School-based delivery programs require health care workers to temporarily suspend their clinic services to conduct community outreach, which was a substantial burden reported among stakeholders. Additionally, healthcare workers are usually not compensated for vaccine outreach, which can deter healthcare providers from participating: *“One of the things I’m learning is how important the health system strength [is] in general…for HPV when we think about service delivery and costs and efficiencies in that system*, *what we see is a lot of countries in Southeast Asia*, *they might have stronger health systems in the sense of human resources*, *and healthcare workers*. *And they may not have to pay as much or as often to make sure that health care workers are going out to do their outreaches to go to the schools “-* Global Immunization Partner

#### Immunization programming and delivery

Oftentimes, immunization programs in many countries largely focused on immunization of infants and young children, resulting in difficulty in developing strategies for targeting older age groups such as adolescents. Reaching this age group, coupled with the need for multiple doses, resulted in difficulties in meeting coverage targets particularly for the second dose: “*There are different reasons for [low coverage]…most of the programs in lower-middle-income countries are geared towards immunizing infants*. *They even have lower coverage with the what we call second year of immunization that measles*, *second dose*, *for example*. *The HPV vaccination schedule is an issue*. *Currently*, *it’s a two-dose schedule*, *and the second dose has to be provided 6 to 12 months after the first*. *And this is quite a long period*…”–Global Immunization Partner. This sentiment was also voiced by another participant: *“In general*, *coverage is highly variable*, *some countries reach relatively high like 70%*, *80%*. *When we look at the 2020 data*, *some reach a moderate coverage usually around the 40%*, *60% …not enough to see an impact*. *That is*, *of course with the second dose*. *There are different reasons for that*, *most of the programs in lower-middle-income countries are geared toward immunizing infants*.*”*–Global Immunization Partner.

The need for newer delivery platforms for HPV vaccines compared to traditional childhood vaccines was described as a hindrance for immunization programs: *“HPV is a newer platform*. *So it does take that extra work from the system*, *and I think I think that’s challenging*. *What do you think the other thing is*, *it’s like*, *it’s not just*, *you know*, *it’s immunization*, *like*, *HPV vaccine is obviously the immunization system*. *But Cervical cancer is like*, *other parts and like*, *I feel that there’s this opportunity to [for HPV vaccines] to be integrated and synergize*. *But I also feel that often it’s really confusing how to do that well*.*”-* Global Immunization Partner.

#### Program impact

Ensuring that appropriate monitoring and surveillance systems were in place to evaluate impact, particularly in countries that had introduced at a sub-national level, was important in supporting the subsequent scale-up of HPV vaccines. Several informants also reported on the importance of surveillance systems for monitoring adverse events: *“There are some supports coming from the international organizations on how to have a robust system of surveillance*, *because with a new vaccine*, *they’re [countries’] having the surveillance system improved is very important…not only just to make sure that the coverage is high*, *but also to keep tabs on the adverse events that are happening*.*”*—Global Immunization Partner.

#### Vaccine accessibility, equity, and ethics

Participants noted barriers to vaccine accessibility relating to socioeconomics, gender, and geography. In countries where vaccines are only available in the private sector, the cost of vaccines is a major barrier. In these scenarios, even when patients are open to receiving the vaccine, the associated costs can be a deterrent:

*“One aspect is the cost because majority will accept [the HPV vaccine]*, *but then when I came back to our hospital*, *and I went to the public health unit*, *where they give those vaccines* …*because it is still not part of our National Immunization coverage…so it’s part of those vaccines that people have to pay out of their own pockets*… *and people’s level of economic status is very low*, *then even when they want to take it*, *the priority is mainly to do with other demands*, *and [they] ignore the issue of HPV vaccine*. *Because at the time*, *the last time I checked …someone will have to spend 10,000 Naira roughly*, *that is about $20…this individual’s entire monthly income is $30–40 per month… and so it’s practically impossible for them*.*”*

–Academic Researcher

The participant also spoke about the cost-prohibitive nature of the vaccine in the private sector: *“For many of the African countries*, *the vaccine might be there in the private sector*. *But again*, *it’s at a cost*. *And this can be a huge deterrent in terms of accessing the vaccine …the limitations in terms of access*, *and a lot of it comes from where the vaccine is not available nationwide*, *to the public*. *And that is for the majority of Sub-Saharan Africa right now*. *This can really be a determining factor in whether or not a parent needs to access an out-of-pocket from the private health sector*.*”–Academic Researcher*

Vaccine equity was mentioned as critical for scale-up, especially the importance of vaccinating boys. Stakeholders reported that in many instances, communities questioned why girls are targeted for vaccination instead of boys, which may lead to skepticism, hesitancy, and decreased uptake. This participant described concerns raised in Rwanda related to this issue: *“[There was] kick back about why are we vaccinating girls*, *not boys …that suspicion of ‘why’ and this was 10 years ago*. *But I think there’s probably*, *unfortunately*, *a lot more vaccine skepticism now …there was a bit of an issue of gender like*, *‘why my daughter and not my son*? *so what are you giving my daughter*? *Is it bad*?’”–Academic Researcher

### Downstream determinants to introduction and scale-up

Downstream determinants were defined as community-level factors that influenced successful vaccine introduction and scale-up, including vaccine acceptability and hesitancy and communications, advocacy, and social mobilization (Table 2).

#### Vaccine acceptability and hesitancy

Stakeholders mentioned that vaccine hesitancy was an issue prior to COVID-19 and had increased during the pandemic. Stakeholders perceived resistance and uncertainty asare high in communities with limited knowledge and awareness about the HPV vaccine, and misinformation was found to be partly driven by social media. As HPV and cervical cancer are seen as a women’s reproductive health issue that is considered taboo in many settings, the topic is unfortunately linked to significant stigma, which in turn perpetuates misinformation. Stakeholders provided examples of how the stigma surrounding cervical cancer also contributes to avoidable deaths, as women were afraid to undergo prevention measures or seek treatment. As one interviewee said, *“Culturally*, *socially*, *socially*, *a lot of females find it discomforting*, *to freely communicate*, *maybe now we are getting some changes with more awareness and people becoming more informed*, *but they find it difficult to freely communicate on anything that has to do with their genital tract*. *And that is part of the reasons why women don’t even present early to the hospital*, *even when they have features like vaginal discharge*, *bleeding*, *contact bleeding…they tend to hide that even from their closest persons until it has reached an advanced stage before the present*.*”*- Academic Researcher

It was emphasized that many communities have low awareness of cervical cancer and its prevention. Stakeholders voiced difficulty building awareness for the HPV vaccine given the slow progression of cervical cancer. An academic stakeholder based in Nigeria compared HPV vaccines to polio vaccines, describing how Nigeria has experienced significant challenges in increasing vaccine acceptability for more visible diseases like polio. They questioned how demand can be generated for a vaccine targeting a disease that is stigmatized and relatively invisible. *“You know*, *polio*, *is a viral infection*, *and a virus of global public health importance*. *People introduce some conspiracy*, *that made people refuse accepting polio vaccines at a point…and there were a lot of insinuations that this is a vaccine that was primarily introduced just to control people’s fertility*. *So what do you expect when you now introduce another vaccine*? *And you say this one is*, *is to prevent you from getting an infection*? *And you’re not just talking about any infection*, *but you’re picking an infection right in the womb*?*”*–Academic Researcher, Nigeria

Rumors have added to vaccine hesitancy. One participant discussed this issue in the context of polio: *“You know*, *polio is a viral infection*, *and a virus of global public health importance*, *and the whole world is attempting to eradicate polio… People introduce some conspiracy*, *what I can call conspiracy theories*, *that made people refuse polio vaccines at a point…And there were a lot of insinuations that this is a vaccine that was primarily introduced just to control people’s fertility*, *and in the process also control their reproduction and population*. *So*, *what do you expect when you now introduce another vaccine*? *And you say this one is to prevent you from getting an infection*? *And you’re not just talking about any infection*, *but you’re picking an infection right in the womb*?*”*–Academic Researcher

Another stakeholder emphasized that acceptability is especially challenging for adolescent vaccines: *“People are convinced [about childhood vaccines] … people understand children should be vaccinated*. *You don’t need to convince them they understand that childhood diseases have killed people*. *But now introduce a vaccine to an older age group…they’re like*, *‘What*? *Why*? *Is it safe*?*’ And then add that to social media negativity…”*–Academic Researcher

Several stakeholders stressed that vaccine hesitancy is widespread not only among adolescents and parents, but also among policy makers, educators, and health care providers: *“…Some of the advice not to get vaccines comes from health professionals themselves*. *Some of them were taken as policymakers*. *And some of them were actually academicians*, *some of them were even involved in research on vaccines… how can we actually really increase acceptance for Western medicine*, *given the dark history of colonialism and medicine itself*? *So*, *unfortunately*, *we can’t deny that there have been bad episodes in the history of medicine*, *and I think those stories still linger and contribute to the resistance of some of the well-intended process right now*.”–Academic Researcher

Stakeholders provided examples of how in certain instances, health care providers spread misinformation: “*… the most important [consideration] here in this region*, *is this idea that this vaccine is not safe*. *And most importantly*, *they [ideas that the vaccine is not safe] are shared by medical workers*. *And I think it’s the major problem…because if medical workers were sure that it’s a good vaccine then I think all these rumors on the internet and social media wouldn’t work…all studies show that people trust medical workers*, *but if medical workers are hesitant then it’s a big problem…”-* Global Immunization Partner

#### Communications, advocacy, and social mobilization

Communications efforts remain critical for addressing vaccine hesitancy and creating demand for HPV immunization programs. All participants emphasized the need to increase funding for communications, both during vaccine introduction as well as post-introduction. They provided examples of when communications funding was present during the initial phases of vaccine introduction but dwindled afterward: *“When we launched the HPV vaccine*, *the President was on TV and on radio*. *Newspapers were on it*. *But the funding to continue community education isn’t there…”*–Academic Researcher

Stakeholders expressed concerns about ensuring that communications efforts are streamlined and consistent. In several countries, messages about the safety and efficacy of the HPV vaccine have been unclear. Additionally, academic stakeholders in Kenya, South Africa, Zambia, and the UK, as well as several global immunization partners, described how messages emphasizing the sexual transmission of HPV have been highly ineffective. Stakeholders described the need to transform communications initiatives and ensure messages are aligned with countries’ cultural conventions: *“The risk communication strategy initially was under development…the value of it was under appreciated…the standard of the government in terms of the efficacy and safety of the vaccine has to be very consistent*. *The moment there is confusion in terms of the messaging…this communication approach is something that has to be much more structured*, *much more planned*, *and it has to be planned even before the vaccine is introduced*. *So that way*, *we can take care of these kinds of problems that may happen*.*”-* Global Immunization Partner

One participant discussed the need for sustained communication and education. *“I think there’s been an under investment*. *I think that’s one of the things that was taken for granted*, *something that we picked up in pre-introduction*, *and then again*, *at the time of introduction*, *is that there will be a constant need to communicate about the value of vaccination*, *but also*, *you know*, *people had difficulty understanding the link between HPV and cervical cancer*, *so just basic health education and promotion about what we’re trying to achieve*. *And I think that*, *if there are resource constraints*, *the first thing that gets cut is the communication budget*.”—Academic Researcher

Global immunization partners indicated that many countries employed communications strategies that worked for other vaccines but that were ineffective for HPV vaccines. This participant described issues related to message framing: *“The worst mistake that countries did is to link this communication campaign with any kind of sexual education with any kind of like getting into this sexual transmission*, *pointing out vaccination before sexual debut*, *I think it was really wrong*. *At least it didn’t work in our region*, *then parents link the sexual behavior with vaccination*, *and decided that their girls do not need this vaccine*.”—Global Immunization Partner

Stakeholders described potential strategies to improve communications efforts: *“Now*, *there’s a lot of room for improvement on the communication*, *social modernization front*, *starting much earlier using much diverse channels and increasing the overall funding and attention to these components will help a lot to create awareness among the public*, *and then to ensure the acceptance and the uptake of the vaccine*. *Currently*, *it’s not optimally done…there’s a lot to do in the training and also addressing people’s questions or concerns*. *Also*, *we can do better in using the adolescent themselves as the change agents who can be empowered to establish their own opinion and also influence the decisions of their caregivers and communities*…”–Global Immunization Partner

Stakeholders described that in many instances, governments used strategies that were successful for traditional childhood vaccines but generally did not translate into success for HPV vaccines. The need to generate evidence and lessons learned from past HPV vaccine communications strategies to inform the development of future communications interventions was suggested: *“We know now*, *decades of implementation of HPV vaccines*, *what the levers are in terms of the messaging*, *and how parents need to get those messages and from whom*. *[It’s hard]… to implement a communication strategy that targets just that*, *because they’re [countries] used to doing it a certain way*. *So*, *it’s really hard*, *this sort of institutional inertia*, *and you’re working up against that*, *in trying to convince governments to not repeat the mistakes of others that you have seen*…”–Global Immunization Partner

## Discussion

This study highlights global perspectives regarding the upstream and downstream challenges of the implementation and scale-up of HPV vaccination programs within LMICs through interviewing academic and NGO stakeholders. Over the past decade, HPV vaccination efforts in LMICs have gained momentum, largely due to the inclusion of HPV vaccines in the Gavi portfolio [35, 36]. However, expansion of HPV vaccine programs in LMICs has been subpar compared to HICs. Our results confirm that strong political commitment and governance are major factors for successful HPV vaccine introduction and scale-up, with similar findings reported in previous studies [37–39]. The patronage of influential figures (i.e., First Ladies) was essential in the promotion of HPV vaccines, as cited in prior studies in sub-Saharan Africa [35, 40, 41]. Upstream challenges related to financing and capacity were paramount, even in countries with Gavi support. Stakeholders in our study highlighted financial challenges beyond vaccine pricing, such as the additional resources required to deliver the HPV vaccine. These financial challenges led numerous stakeholders to voice concerns regarding program sustainability, which was also seen in previous studies (13,34). Concerns about program sustainability are especially relevant in Gavi transitioning countries, which often struggle to secure domestic resources to fund immunization services [42]. The current manufacturing cost of the quadrivalent HPV vaccine is significantly lower than the Gavi purchase price. Given the manufacturing costs, there may be opportunities for further negotiation on reducing the Gavi purchase price, with the acknowledgement that this would require careful negotiations [21, 43]. New manufacturers on the market (i.e. Serum Institute, CERVAVAC) are committing to increasing production of the HPV vaccine supply with the potential to vaccinate up to 84 million adolescent girls in Gavi-eligible countries by 2025 [44]. As new manufacturers enter the market, it will be imperative for global entities to provide aid to all LMICs- including those that are Gavi ineligible. Priority must be placed on developing strategic funding arrangements and providing access to HPV vaccines at a lower cost for countries that have yet to introduce [44]. Future research should investigate procurement procedures and the role of stakeholders including manufacturers in the procurement process, and evaluate procurement mechanisms to maintain vaccine equity, accessibility, and high coverage rates [45].

Stakeholders in our study emphasized the efficacy of school-based delivery platforms and integrated adolescent health services. HPV vaccine demonstration projects in LMICs have indicated that school-based delivery significantly increases vaccine coverage compared to delivery in a health facility [6, 46, 47]. However, the financial requirements needed to sustain school-based delivery can be monumental. There is a need to champion the allocation of critical resources while maintaining cost-effectiveness in HPV vaccine introduction and scale-up [15, 27].

Our study described additional upstream barriers related to supply constraints which contribute to challenges in the introduction and scale-up of HPV vaccines. HPV vaccine supply constraints have affected delivery targets, as the global HPV vaccine supply cannot meet the aggregate demand [48]. This has caused Gavi-supported countries to shift from multi-aged cohort delivery to single-aged cohort delivery [15]. Supply constraints also hamper non-Gavi countries, as they are less able to negotiate favorable vaccine prices [15]. Recently, there have been sizeable efforts in increasing manufacturing capacity to expand vaccine supply [15, 49, 50]. Increasing the global supply of HPV vaccines may heighten equity in vaccine accessibility [15, 49]. In the face of current supply challenges, it is important to highlight the recent SAGE recommendation which points to the efficacy of a one-dose HPV vaccine schedule [51–55]. This recommendation will be pivotal in vaccine scale-up by increasing vaccine supply and reducing associated resources and costs. Increased supply can potentially catalyze large-scale coverage for different age cohorts and for children of all genders [15].

As more countries introduce HPV vaccines into their national immunization programs and develop plans for scaling up vaccination efforts, strategic approaches in communications, advocacy, and social mobilization must be developed to increase vaccine uptake [56–59]. This is especially relevant as stakeholders indicated that vaccine hesitancy was a substantial barrier to meeting HPV vaccine coverage targets. Misinformation disseminated through social media platforms, negative media reports, anti-vaccine sentiments, and societal stigma has been widely reported in LMICs [15, 41, 43, 60–64]. Efforts to enhance advocacy and communications will be essential to increase the demand for HPV vaccines and raise cervical cancer as a pressing public health issue. Advocacy and communications efforts are needed to not only target adolescents and parents, but to also target healthcare providers, educators, and policymakers. Communications must be broad-based and accessible to influence knowledge, beliefs, attitudes, and practices toward HPV vaccines, as this will be critical in expanding vaccine uptake [65].

### Limitations

This study has several limitations. Our sample was small, however we aimed to maintain diversity in our sample which was demonstrated by our stakeholders being based in several different settings. While it would have been beneficial to triangulate our findings with that of national perspectives such as Ministry of Health personnel, our focus in this manuscript was on obtaining data from global stakeholders as their perspectives are not as readily highlighted in the literature. Furthermore, the majority of stakeholders that were included had experience within sub-Saharan Africa, with fewer participants having experience in other regions which is another limitation of our study. Social desirability bias could have influenced responses. Although the authors developed a comprehensive strategy to identify global stakeholders for this study, it is possible that the search strategy was limited and did not identify all relevant stakeholders. Furthermore, several individuals who demonstrated interest in participating in our study were lost to follow-up, which may have contributed to selection bias.

## Conclusions

Overcoming barriers to HPV vaccine introduction, scale-up, and sustainability and taking sector-wide approaches will be necessary to meet cervical cancer elimination goals [13, 37, 42, 60, 64, 66]. Countries must have access to the best available evidence to facilitate political support for the introduction of HPV vaccines. Additionally, global stakeholders must collaborate with countries to consider mechanisms to reduce financial and capacity challenges to promote the long-term sustainability of vaccination programs. Taken together, our findings indicate a need for continuous engagement between key stakeholders to facilitate the development of evidence-driven, adolescent-centered strategies to increase HPV vaccine scale-up by enhancing access, equity, communications, advocacy, and social mobilization while reducing vaccine hesitancy. Efforts must be placed in ensuring that stakeholders recognize cervical cancer elimination as a worthwhile investment that should be prioritized. Strategies must be developed to support immunization efforts in LMICs with overstretched public health systems and limited health budgets [67]. Vaccination remains crucial to achieving cervical cancer elimination goals, as screening and treatment require a strong health care infrastructure and immense human resources which are widely unavailable in many low-resource settings. This positions HPV vaccination as the most efficacious approach for large-scale reductions in cervical cancer disease burdens [44].

## Supporting information

S1 File Key informant interview guide: Global stakeholders. (DOCX)
