# Supplementary material for: Global perspectives of determinants influencing HPV vaccine introduction and scale-up in low- and middle-income countries
Source: PLoS One. 2024 Jan 16;19(1):e0291990. doi: 10.1371/journal.pone.0291990 (PMC10791006; doi:10.1371/journal.pone.0291990)
Supplement: S1 File — (DOCX) [file pone.0291990.s001.docx]

**Key Informant Interview Guide: Global Stakeholders**

This interview aims to better understand the decision-making processes that take place among key stakeholders (Global immunization partners, academic stakeholders) in the introduction and implementation of HPV vaccination programs in LMICs. Data obtained from this research will be used to support policy decision making for HPV vaccine implementation moving forward.

The information you provide will be combined with information from other people and relevant documents to develop reports, manuscripts, and inform future workshops. We won’t use your name and would just refer to whether you are a global or national stakeholder and your country/geogrpahic region

**Prioritization of HPV vaccination**

*Let’s first talk about how cervical cancer prevention and HPV vaccination are prioritized relative to public health and vaccination programs within LMICs.*

1. Tell me about yourself - current position, organization, role/responsibilities with your org, etc.

**Challenges to HPV Vaccine Introduction and Scale-Up**

1. Briefly, what are the biggest obstacles facing HPV introduction in LMICs?
   1. Are there global challenges? Are there region-specific challenges?
   2. What about where you work or have experience?
   3. Probes: Supply constraints, delays due to COVID-19, research challenges (e.g., one-dose issue), political commitment, demand or vaccine hesitancy issues
2. Challenges in allocating national health budget to implementing and sustaining actual HPV vaccination program
3. What is being done and by whom about these challenges at the global and national level?
   1. Are these efforts succeeding?
   2. What timeframes do you envision for their success?
   3. What more should be done?
4. What is working and why? Where specifically and why in this context?

**Prioritization and decision-making around HPV vaccination**

1. Tell me about how the vaccine prioritization process unfolds in the countries you have worked in.
   1. Where would you say costing data falls as a motivator for HPV vaccine introduction
   2. What are the biggest motivators for prioritizing HPV vaccination globally and nationally?  Ask about: Perceptions of the health problem, disease burden, intervention efficacy, finances, health system capacity, demand, costing data
   3. How is HPV vaccine introduction prioritized given the competing health priorities in many countries?
   4. What are the biggest demotivators or detractors to prioritization of HPV vaccination? Ask about: Gender biases (because it’s a women’s health issue), long time scale from infection to disease onset, shame/stigma, adult disease (not childhood)
2. What factors are considered in the prioritization of HPV vaccination programs compared to other vaccines?
   1. In the sector of women’s health, how is HPV vaccination and cervical cancer prevention prioritized?
3. In your experience, what would you recommend to improve prioritization and decision-making of HPV vaccination?
   1. Globally?
   2. Nationally?
   3. How can the key actors you mentioned be best engaged or supported in decision-making about HPV vaccination?

**Evidence-based decision-making processes**

*Let’s discuss how decisions are made around HPV vaccination globally and nationally.*

***Global Relevant decision-making ( Academia, Global immunization partners)***

1. Describe the role of global partners in the decision-making process for HPV vaccine introduction?
2. Describe how global partners coordinate with national stakeholders during the decision-making process?
   1. What guidelines or criteria are used among global stakeholders for country selection in introducing HPV vaccination?
3. Describe the influence of external vaccine support (e.g. GAVI Vaccine Alliance) in driving vaccine adoption decisions
4. Given the global challenges that you previously identified (manufacturing shortages, COVID-19 delays, etc.), what is being done and by whom to address these challenges?
5. What policy or programmatic gaps related to challenges do you see at the global level? How could they be filled and by whom?

***Vaccine Delivery***

*The next few questions will focus on HPV vaccine delivery along with public acceptability of HPV vaccines and how these influence the decision-making process.*

1. HPV vaccination programs are often packaged with other adolescent health services to enhance vaccine delivery.? (e.g. GAVI Alliance integrating age-relevant services such as deworming and nutritional supplements, integrating HPV vaccination with sexual and reproductive health education). How are packages decided upon?
   1. Is integration of HPV vaccination with other adolescent health services considered in the decision making process in your country (countries that have introduced HPV vaccination programs)? or will be considered ( countries that are planning to introduce)
   2. What are the reasons that integration of HPV vaccination programs with other

adolescent health services is considered/not considered?

- 1. Are countries provided an opportunity to voice or select which services to prioritize in this package ? Are services selected without the input of in-country stakeholders?

**Vaccine Hesitancy**

1. Is vaccine acceptance or hesitancy a major issue influencing HPV vaccine introduction and scale-up?
   1. Globally? Regionally? Nationally?
   2. At what stage of the decision-making process is assessing public acceptance towards HPV vaccines considered?
   3. Describe challenges that have occurred in the acceptability of HPV vaccines among the general public. How have such challenges been addressed (e.g. increasing education, mass communication and media campaigns to heighten awareness, community engagement) ?

**Scale up of HPV Vaccination During the COVID19 Pandemic**

*We would like to ask a few questions about the impact of COVID-19 on the scale-up of HPV vaccination.*

1. How has COVID-19 impacted HPV vaccine introduction and scale-up? How will this play out over the next 12 months?
   1. Describe how the COVID-19 pandemic has affected strategies for the delivery of HPV vaccines (e.g. school closures affecting HPV vaccine delivery) ?
2. How has HPV vaccination remained a priority for high-burden LMICs in the context of the COVID-19 pandemic?

a. Has the prioritization of HPV vaccines (and other vaccines) been diverted to COVID-19 vaccines during the pandemic?

b. Are there any additional factors that now are considered in HPV vaccine introduction which were not a concern prior to the COVID-19 pandemic?

1. Has HPV vaccine hesitancy, mistrust, or misinformation increased during the COVID-19 pandemic?

**Other**

1. What have I not asked that you think is important for us to know?

1. What other information do you think would be helpful to us to consider about the decision-making process? (additional comments, names of other people to interview, documents)
